# Gynecological morbidity and treatment-seeking among older adult (aged 45–59) women in India

**DOI:** 10.1186/s12978-023-01611-1

**Published:** 2023-04-27

**Authors:** Manas Ranjan Pradhan, Sourav Mondal, Prasanna Kumar Mudi

**Affiliations:** 1 Department of Fertility and Social Demography, International Institute for Population Sciences, Govandi Station Road, Deonar, Mumbai, 400088 India; 2 Department of Fertility and Social Demography, International Institute for Population Sciences, Mumbai, India

**Keywords:** Gynecological morbidity, Treatment-seeking, Older adult women, India

## Abstract

**Background:**

Women’s gynecological health needs are not limited to the reproductive years of their life. Women are at risk of hormonal changes, gynecological malignancies, and various genitourinary conditions as they move toward menopause and beyond. Concerns about older women’s sexual and reproductive health and rights (SRHR) continue to be taboo in many countries, of little interest to researchers and professionals in the field of healthcare, and a “blind spot” in discussions about policy as a whole. Despite the widespread agreement, the life course approach to addressing SRHR concerns has received minimal attention. The study estimates the prevalence, assesses the correlates, and treatment-seeking of gynecological morbidity (GM) among older adult women aged 45–59 years (N = 18,547) in India.

**Method:**

The analysis was based on the nationally representative Longitudinal Ageing Study (2016–2017) data that adopted a multistage stratified area probability cluster sampling to select respondents. The outcome variables used in this analysis were ‘had any GM’ and ‘sought treatment for any GM.’ Women with any morbidity such as per vaginal bleeding, foul-smelling vaginal discharge, uterus prolapses, mood swings/irritability, fibroid/cyst, and dry vagina causing painful intercourse were considered to have any GM. Of the respondents with GM, who sought a doctor’s consultation or treatment were considered ‘sought treatment for any GM.’ Binary logistic regression was conducted to examine the adjusted effect of socioeconomic and demographic predictors of GM and treatment-seeking. Stata (V 16) was used for statistical analyses with a 5% significance level.

**Results:**

Fifteen percent of the women had any GM, and only 41% of them sought treatment. Age, marital status, education, number of pregnancies, hysterectomy, involvement in household decision-making, social group, religion, wealth status, and region were significantly associated with GM. The odds of treatment-seeking were higher among women with 10+ years of schooling (OR 1.66, CI 1.23, 2.23), with hysterectomy (OR 7.36, CI 5.92, 9.14), with five-plus pregnancies (OR 1.25, CI 0.96, 1.64), and those from the richest (OR 1.91, CI 1.40, 2.60) households than their respective counterparts.

**Conclusion:**

Many older adult women experience GM, and treatment-seeking is inadequate. The GM prevalence and treatment-seeking vary considerably by socioeconomic and demographic characteristics. Results suggest community-level awareness generation and the inclusion of this otherwise ignored group in programs targeting better health and wellbeing of women.

## Introduction

Gynecological morbidity (GM) includes any condition, diseases or dysfunction of the reproductive system which is not related to pregnancy, abortion or childbirth, but may be related to sexual behavior [1]. Gynecological problems are major causes of illness and mortality worldwide, with women in Lower- and Middle-Income Countries (LMICs) bearing the majority of the disease load. The gynecological disease makes up 4.5% of the global disease burden, more than other global health concerns like malaria, TB, ischemic heart disease, and maternal diseases [2]. Women’s gynecological health needs are not limited to the reproductive years of their life. Women from LMICs experience GMs throughout their reproductive years and beyond, in part due to the limited medical care they receive during labor and delivery, combined with high parity [3]. Moreover, the sexual health of older women is often considered taboo in many cultures [4], including India. Studies on the sexuality of older ages in the Indian setting indicate the prevalence of sexual activities among middle-aged heterosexual couples [5, 6]; nevertheless, public discourse on the subject is avoided to prevent unfavorable cultural attitudes [7].

Women are at risk of hormonal changes, gynecological malignancies, and various genitourinary conditions as they move toward menopause and beyond [8]. The lining of the vagina can reduce innate protective mechanisms against infection among postmenopausal women, and older women having chronic pelvic infection plus reduced immunity are vulnerable to different infectious diseases, including HIV [9]. Evidence suggests women themselves may not seek care, often because they accept the physical discomforts associated with gynecological problems, menopause, and aging as natural [10].

In India, the 2017 National Health Policy (NHP) envisages as its goal the attainment of the highest possible level of health and wellbeing for all at all ages, through a preventive and promotive health care orientation in all developmental policies and universal access to good quality health care services without anyone having to face financial hardship as a consequence. More specifically, the NHP targets enhanced provisions for reproductive morbidities and health needs of women beyond the reproductive age group (40+) [11]. India Strategy for Women, Adolescents and Child Health (I-WACH) builds on these policies to articulate a life-course approach to women’s health [12]. The life cycle approach to providing health services, including sexual and reproductive health (SRH) services, refers to offering services over the course of a client’s life, making sure that women’s SRH needs are met all throughout their lives [13]. Nevertheless, concerns about older people’s SRH and rights (a) continue to be a taboo topic and (b) of little interest to researchers and professionals in the healthcare field. Despite the widespread agreement, the life course approach to addressing SRH and rights has received minimal attention [14–17]. Moreover, as India moves closer to Universal Health Coverage, it is important to assess if policy initiatives to broaden women’s health beyond maternal health and family planning have increased women’s service utilization.

In India, the prevalence of GM ranges between 43 and 92% [18–21]. Most of the literature on GM in the Indian context cover women of reproductive age (15–49 years); and years of schooling, age, religion, caste, number of pregnancies, autonomy, mass-media exposure, economic status, and place of residence were found to be the significant predictors of GM and treatment-seeking [18, 22–25]. However, the GMs of postmenopausal women has received minimal attention so far as policy focus and research is concerned, though there is some attention on their general health [26]. Existing scanty evidence on GM and treatment seeking of older adults in India are based on small-scale community-level studies [18]. Against this backdrop, using a nationally representative sample, the present study estimates the prevalence and assesses the determinants and treatment-seeking of GM among older adult women (45–59 years) in India. Results will be a benchmark for assessing women’s reproductive health undergoing premenopausal/menopausal transition in India.

## Methods

### Data

**Fig. 1:**
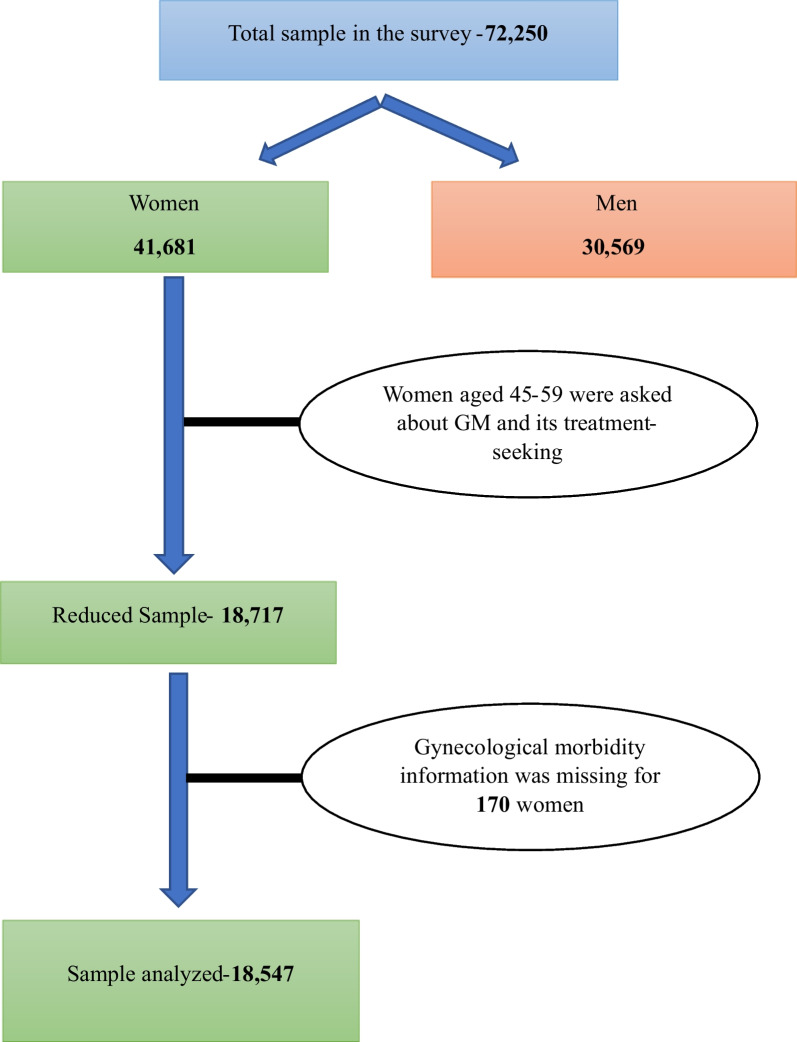
Flowchart of sample selection

The study used data from the Longitudinal Ageing Study in India (LASI-Wave 1), 2016–2017. The International Institute for Population Sciences (IIPS), the Harvard T.H. Chan School of Public Health, and the University of Southern California collaborate to conduct the LASI, a multi-topic, nationally representative, large-scale survey. It offers crucial details on chronic illnesses, symptom-based illnesses, demography, functional and mental health, household economic status, healthcare utilization, health insurance, work, employment, and retirement, as well as life expectations for participants 45 years of age and older with their spouses. The LASI is a nationally representative survey covering 72,250 individuals aged 45 and above and their spouses. The study adopted a multistage stratified area probability cluster sampling design to select the observation units, i.e., older adults aged 45 and above and their spouses, irrespective of age. Trained research investigators gathered the data using computer-assisted personal interviewing (CAPI). Only those respondents who gave oral/written consent were interviewed in the survey. The published national report provides detailed survey design, questionnaire, and quality control measures [27]. The survey asked female respondents aged below 60 about GM and its treatment-seeking. Of the 18,717 surveyed women aged 45–59 years, information on GM was missing for 170, and thus data from 18,547 women were finally considered for analysis (Fig. 1).

### Outcome variables

The outcome variables used in this analysis were ‘had any GM’ and ‘sought treatment for any GM.’ In the survey, women below 60 were asked, ‘in the last 12 months, have you had any of the following health problem(s)?’. The respondents with any enquired GMs such as per vaginal bleeding, foul-smelling vaginal discharge, uterus prolapses, mood swings/irritability, fibroid/cyst, and dry vagina causing painful intercourse were considered to have any GM. Women with any of the symptoms above were further asked, ‘did you seek a doctor’s consultation or treatment for any of these health problems?’. Women responding ‘yes’ to this question were considered to have sought treatment.

### Predictor variables

The individual characteristics such as current age (45–49, 50–54, 55–59 years), marital status (currently married/others), years of schooling (no formal education, < 10 years, 10+ years), number of pregnancies (< 3, 3–4, 5+), hysterectomy (yes, no), mass media exposure (full, partial, never), and involvement in any household decision-making activities (yes, no) were included in the analysis to assess the role of the women’s characteristics in the prevalence and treatment-seeking of GM. Health insurance (yes, no) was included as an additional predictor variable for treatment-seeking of GM. Additionally, the household features like social groups (scheduled caste-SC, scheduled tribe-ST, other backward classes-OBC, Non-SC/ST/OBC), religion (Hindu, Muslim, others), and Monthly Per Capita Consumption Expenditure (MPCE) (poorest, poorer, middle, richer, richest) and community-level characteristics such as residence (urban, rural) and geographical regions (north, central, east, northeast, west, south) were included in the analysis to assess their association with GM and treatment-seeking. MPCE was computed using data on consumption expenditure collected using the abridged version of the National Sample Survey (NSS) consumption schedule. Women reading newspaper/watching television daily or several times a week were considered to have full mass media exposure, while those reading newspaper/watching television sometimes or rarely were considered to have partial exposure, and those women who never had read newspaper/watched television were considered to have no mass media exposure. Women’s involvement in household decision-making was assessed through their participation in paying bills and settling financial matters, advising the children, and settling disputes. In the survey, women were asked, “Are you usually involved in the following household activities, such as cooking, shopping for the household, payment of bills and settling of financial matters, taking care of household chores, giving advice to the children, settling disputes, and other decisions?”. In this analysis involvement of women in any of the three activities above, assumed to be crucial measures of autonomy, was considered.

### Statistical analysis

The descriptive statistics of the study population by selected socioeconomic and demographic characteristics of the women have been presented for the sample considered for analysis. Additionally, as the outcome variables were dichotomous, binary logistic regression was employed to examine the adjusted effect of socioeconomic and demographic predictors of GM and treatment-seeking of older adult women. The predictor variables included in the regression analysis were finalized after assessing their independent association with the outcome variable (any GM) and checking collinearity among the predictor variables. Multicollinearity was evaluated through Variance Inflation Factor (VIF) method. National individual sample weight was used in the analysis. LASI sample weight accounts for selection probabilities and is adjusted for nonresponse and post-stratification to represent the population characteristics accurately. Stata (V 16) was used for statistical analyses with a 5% significance level.

## Results

### Socioeconomic and demographic profile of the older adult women

Table 1 presents the socioeconomic and demographic characteristics of the surveyed women aged 45–59 years. Of the women, 39% were 45–49 years old, 31% were 50–54 years old, and the rest were aged 55–59 years. Nearly four out of every five women (79%) were currently married. About three-fifths (57%) of the women had no formal education. Of the women, 26% were pregnant less than three times, 41% were three to four times, and the rest had five or more pregnancies. Thirteen percent of these women had undergone hysterectomy. More than half (53%) of the women had full mass media exposure, one-fifth of them had partial exposure, and the rest (26%) had no mass media exposure. Nearly four-fifths (79%) of the women were involved in household decision-making. About one-fifth (21%) of the women had health insurance. An almost equal proportion of these women belonged to the MPCE quintiles. Of the total women, 46% were from OBC, 26% from non-SC/ST/OBC, 20% from SC, and 9% from the ST category. A majority (81%) of the women were Hindus. Two-thirds of these women reside in rural areas. Twenty-six percent of the women belonged to the southern region, 23% to the east region, 19% to the central region, 16% to the western region, 12% to the northern region, and 4% to the north-eastern part of the country.

**Table 1:** Socio-economic, demographic and health profile of the older adult women (45–59 years), India, 2017–2018

Characteristics	Percentage distribution	95% CI of all proportions	Number of women
Age			
45–49	39.03	(0.3901366–0.3903737)	7201
50–54	30.95	(0.3094079–0.3096325)	5863
55–59	30.02	(0.3001133–0.300336)	5483
Marital status			
Others	21.46	(0.2144864–0.2146859)	4120
Currently married	78.54	(0.7853141–0.7855136)	14,427
Years of schooling			
No formal education	57.49	(0.5747975–0.5750377)	9832
Up to 9 years	27.75	(0.2774376–0.2776552)	5890
10 and more years	14.75	(0.1474499–0.1476222)	2825
Number of pregnancy^#^			
< 3	25.97	(0.2595695–0.259785)	4831
3–4	41.43	(0.4141432–0.4143853)	7621
5+	32.61	(0.3259434–0.3261738)	5710
Hysterectomy^#^			
No	86.96	(0.8694826–0.8696463)	16,443
Yes	13.04	(0.1303537–0.1305174)	2094
Mass-media exposure^#^			
Never	26.41	(0.2639707–0.2641855)	4455
Partial	20.43	(0.2041531–0.2043495)	3793
Full	53.17	(0.531549–0.5317922)	10,199
Involvement in household decision making^#^			
No	20.51	(0.2050343–0.2052313)	3525
Yes	79.49	(0.7947687–0.7949657)	14,869
Health insurance^#^			
No	78.87	(0.7885915–0.7887904)	14,138
Yes	21.13	(0.2112096–0.2114085)	4301
Social group			
Scheduled caste (SC)	19.74	(0.197278–0.1974714)	3196
Scheduled tribe (ST)	8.93	(0.0891988–0.0893374)	3340
Other backward classes (OBC)	45.80	(0.4578405–0.4580826)	6944
Non-SC/ST/OBC	25.54	(0.2552898–0.2555017)	5067
Religion			
Hindu	81.26	(0.8124662–0.8126559)	13,576
Muslim	11.80	(0.1179583–0.118115)	2293
Others	06.94	(0.0693406–0.0694641)	2677
Monthly Per Capita Consumption Expenditure (MPCE) quintile			
Poorest	20.00	(0.1999149–0.2001092)	3494
Poorer	20.52	(0.2051033–0.2052995)	3659
Middle	20.58	(0.2056818–0.2058782)	3669
Richer	19.73	(0.1972392–0.1974326)	3788
Richest	19.17	(0.1915751–0.1917664)	3937
Place of residence			
Rural	66.08	(0.6606985–0.6609286)	11,783
Urban	33.92	(0.3390714–0.3393015)	6764
Region			
North	12.09	(0.1208419–0.1210003)	3382
Central	19.45	(0.1944261–0.1946185)	2478
East	22.53	(0.225191–0.225394)	3186
Northeast	03.70	(0.0369745–0.0370662)	2442
West	15.85	(0.1584496–0.1586271)	2475
South	26.37	(0.2635985–0.2638126)	4584
Total	100		18,547

^#^Number may not add to total due to missing cases

### Prevalence of GM & treatment-seeking

**Fig. 2:**
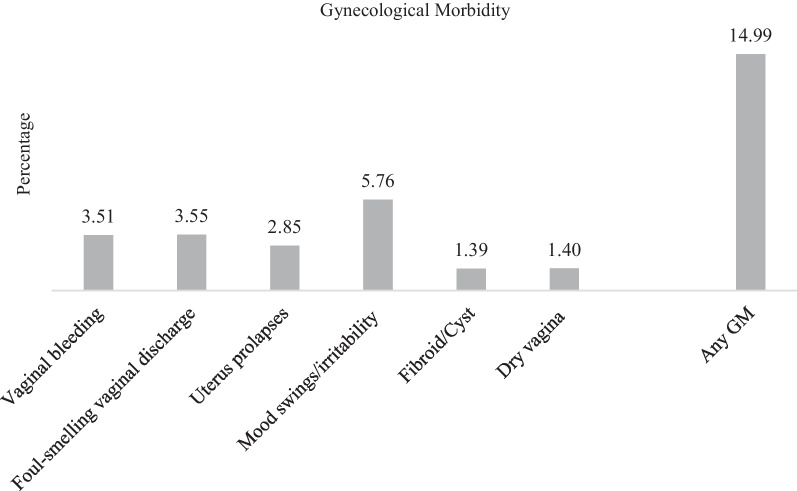
Prevalence of specific gynecological morbidities among older adult women (45–59 years), India, 2017–2018

**Fig. 3:**
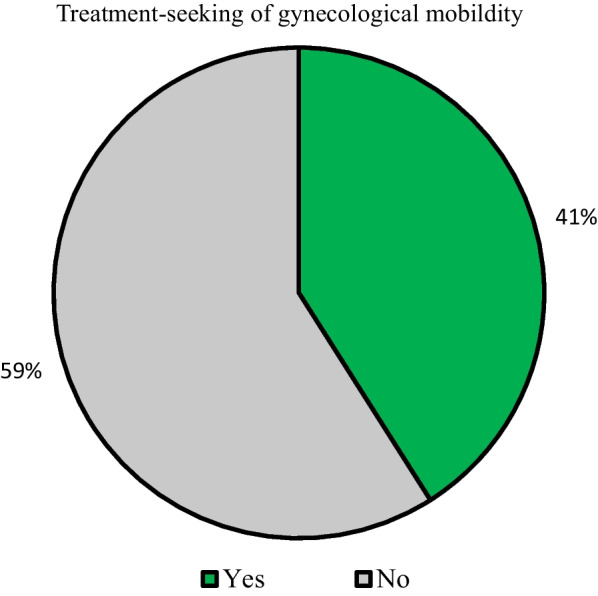
Percentage of older adult women (45–59 years) sought treatment for gynecological morbidity, India, 2017–2018

Fifteen percent of the women aged 45–59 had any GM (Fig. 2). Of them, 6% experienced mood swings/irritability, 4% experienced vaginal bleeding or foul-smelling vaginal discharge, 3% reported uterus prolapse, and 1% reported fibroid/cyst and dry vagina causing painful intercourse. Only 41% of older adult women (45–59 years) had sought treatment for any GM (Fig. 3).

### Determinants of GM & treatment-seeking

After adjusting the effect of other predictors, women aged 50–54 years had 11% (OR 0.89, 95% CI 0.81–0.98), and those aged 55–59 years had 29% (OR 0.71, 95% CI 0.64–0.79) lower odds of having any GM than women aged 45–49 years (Table 2). The probability of any GM was higher among women with any education than their counterparts without formal education. The women with five-plus pregnancies had a 36% (OR 1.36, 95% CI 1.20–1.54) higher likelihood of any GM than those with less than three pregnancies. Women with a hysterectomy were nearly three times more likely to report any GM than those without a hysterectomy (OR 2.96, 95% CI 2.65–3.31). The chances of any GM were higher among women involved in household decision-making (OR 1.30, 95% CI 1.15–1.46) than their counterparts. The odds of any GM were higher among Muslim women (OR 1.22, 95% CI 1.07–1.39) and those Non-Hindu/Muslims (OR 1.26, 95% CI 1.09–1.44) than those Hindu women. Compared with women from SC, the chances of any GM were higher among the women from ST (OR 1.25, 95% CI 1.07–1.46). Women from middle-income households had 27%, those from richer households had 33%, and those from the richest households had 43% higher odds of any GM than those from the poorest households. Compared to the northern region, the likelihood of GM was significantly lower in the southern and western regions (OR 0.66, CI 0.57–0.76) and higher in the central region (OR 1.22, 95% CI 1.05–1.42).

**Table 2:** Adjusted odds ratio (AOR) for gynecological morbidity (GM) and treatment-seeking of the older adult women (45–59 years), India, 2017–2018

Characteristics	GM		Treatment-seeking	
	AOR	95% CI	AOR	95% CI
Age				
45–49^®^				
50–54	0.89**	(0.81, 0.98)	0.9	(0.73, 1.11)
55–59	0.71***	(0.64, 0.79)	0.88	(0.70, 1.11)
Marital status				
Others^®^				
Currently married	1.11**	(1.00, 1.24)	0.87	(0.69, 1.11)
Years of schooling				
No formal education^®^				
Up to 9 years	1.23***	(1.11, 1.36)	1.06	(0.85, 1.32)
10 and more years	1.29***	(1.12, 1.49)	1.66***	(1.23, 2.23)
Number of pregnancies				
< 3^®^				
3–4	1.11**	(1.00, 1.24)	1.03	(0.81, 1.30)
5+	1.36***	(1.20, 1.54)	1.25	(0.96, 1.64)
Hysterectomy				
No^®^				
Yes	2.96***	(2.65, 3.31)	7.36***	(5.92, 9.14)
Mass-media exposure				
Never^®^				
Partial	1.10	(0.97, 1.25)	1.01	(0.76, 1.33)
Full	1.04	(0.93, 1.17)	0.73***	(0.57, 0.94)
Involvement in household decision making				
No^®^				
Yes	1.30***	(1.15, 1.46)	0.85	(0.66, 1.08)
Health insurance				
No^®^				
Yes			0.99	(0.79, 1.24)
Social group				
Scheduled caste (SC)^®^				
Scheduled tribe (ST)	1.25***	(1.07, 1.46)	0.63***	(0.44, 0.90)
Other backward classes (OBC)	1	(0.88, 1.13)	1.12	(0.86, 1.46)
Non-SC/ST/OBC	1.04	(0.91, 1.19)	1.08	(0.82, 1.44)
Religion				
Hindu^®^				
Muslim	1.22***	(1.07, 1.39)	0.78**	(0.59, 1.03)
Others	1.26***	(1.09, 1.44)	0.71**	(0.51, 1.00)
Monthly Per Capita Consumption Expenditure (MPCE) quintile				
Poorest^®^				
Poorer	1.15	(1.00, 1.32)	1.18	(0.86, 1.62)
Middle	1.27***	(1.11, 1.47)	1.14	(0.83, 1.56)
Richer	1.33***	(1.16, 1.54)	1.45***	(1.06, 1.98)
Richest	1.43***	(1.24, 1.65)	1.91***	(1.4, 2.6)
Place of residence				
Rural^®^				
Urban	1	(0.91, 1.11)	1.12	(0.91, 1.39)
Region				
North^®^				
Central	1.22***	(1.05, 1.42)	1.39**	(1.03, 1.88)
East	1.09	(0.95, 1.25)	0.94	(0.7, 1.26)
Northeast	0.84**	(0.71, 1.00)	0.8	(0.52, 1.23)
West	0.66***	(0.57, 0.78)	2.21***	(1.60, 3.06)
South	0.66***	(0.57, 0.76)	1.42**	(1.04, 2.92)

P value: ***Significant at p < 0.01; **Significant at p < 0.05

^®^Reference category

The odds of treatment-seeking were higher among women with 10+ years of schooling (OR 1.66, CI 1.23–2.23), with hysterectomy (OR 7.36, CI 5.92–9.14), with five and more pregnancies (OR 1.25, CI 0.96–1.64), and those from richer (OR 1.45, CI 1.06–1.98)/richest (OR 1.91, CI 1.40–2.60) households than their respective counterparts. Women with full mass media exposure had lower odds (OR 0.73, CI 0.57–0.94) of treatment-seeking than those without any exposure. The chance of treatment-seeking was lower among the STs (OR 0.63, CI 0.44–0.90) than those from SCs. Muslim women (OR 0.78, CI 0.59–1.03) and those Non-Hindu/Muslims (OR 0.71, CI 0.51–1.00) had a lower likelihood of seeking treatment for GM than their Hindu counterparts. The women from the west (OR 2.21, CI 1.60–3.06), central (OR 1.39, CI 1.08–1.88), and south (OR 1.42, CI 1.04–2.92) regions had a higher probability of availing treatment than those from the northern region.

## Discussion

A sizable number of older adult women had GM, and the prevalence varied considerably by their socioeconomic and demographic characteristics. Age of the women, marital status, education, number of pregnancies, hysterectomy, involvement in household decision-making, social group, religion, wealth status, and the region were significantly associated with GM among older adult women.

Higher GMs among women aged 45–49 than those older may be associated with perimenopause/menopause, as evidence suggests that women experience gynecological concerns around menopause [28]. Women experiencing menopausal symptoms had a significantly lower health-related quality of life and higher work impairment than women without menopausal symptoms [29]. Increased GM among women with better education, economic status, and household decision-making indicates better awareness about GM and, thus, reporting. Lower GM among those currently not married women may be due to underreporting as GMs are often perceived to be associated with the sexual behavior of the women. Sexual intercourse beyond marital union continues to be a taboo in India [7], which might influence the reporting of GMs among those not in unions. Thus, there is a high likelihood that women not in marital unions are likely to underreport or not report the GMs to avoid stigmatization. However, given their exposure to sexual intercourse, older women in the union are likely to experience GM such as—a dry vagina causing painful intercourse. Women with autonomy in household decision making are more likely to report GM, while their disadvantaged counterparts may either ignore or perceive GM as natural corresponding to their age and hence not report it [10]. An earlier study also reveals that sexual autonomy is a significant predictor of self-reported sexually transmitted infections (STI) [30]. Religious and cultural beliefs were barriers to accessing SRH services and information among Muslims [31]. Perhaps that explains the higher prevalence of GM among women following Islam. In conformity with earlier community-based studies [32, 33], this study also found a higher prevalence of GM among the STs, which is often credited to their inadequate knowledge about RH and lower utilization of RH care services. The GM adversely affects women’s health and wellbeing, urging program and policy attention. Gynecological morbidities affect women’s physical and psychological life, social role, and religious life [34]. Women with symptoms of GMs have shown an inability to complete their daily routine work [35]. Gynecological problems further affect psychological health [36], and symptom for a longer time is significantly associated with psychiatric morbidity [37].

The study found inadequate treatment-seeking for GM, which conforms with an earlier study [38], which revealed that services for reproductive tract infection (RTI) remain a challenge for women in India. Another possibility may be that women perceive these problems as usual during older ages and are not seeking treatment [10]. Ageism, which refers to age-based stereotypes, prejudice, and discrimination, is another issue that restricts older people’s access to healthcare. Moreover, older people’s SRHR continues to be a taboo topic, affecting treatment-seeking [4, 13]. Untreated RTI can cause cervical cancer and pelvic inflammatory disease [39] and affect psychological life. Evidence suggests thousands of women die from the sequelae of undiagnosed or untreated RTIs, including cervical cancer, ectopic pregnancy, acute and chronic infections of the uterus and fallopian tubes, and puerperal infections [40]. RTIs/STIs also increase the risk of HIV transmission [41].

The study found that higher percentages of women with a hysterectomy are going for treatment, possibly due to hysterectomy-induced GMs and the need for regular health check-ups. Evidence reveals several adverse effects of hysterectomy, such as urinary incontinence [42], sexual dysfunction [43], late medical problems such as backache and weakness [44], and earlier onset of menopause [45]. Women with more pregnancies seek treatment for GM, indicating possible awareness of GM, as most of them were found to have any GM. The inverse association between mass media exposure and treatment-seeking may be because there is insufficient/inadequate information about the GM of older adults in the mass media. The women’s household decision-making autonomy did not significantly influence treatment-seeking. This may be because, besides autonomy in household decision making, treatment-seeking requires resources like money, time, availability of services, and permission from husbands. Hence, it will not necessarily enhance treatment seeking. An earlier study reveals that one-third of those women with GM who did not seek treatment conveyed their problems to their husbands. However, husbands often (a) do not perceive the GM as a problem, thus ruling out treatment seeking, (b) do not feel the need to accompany their wife for treatment, and the wife cannot go alone to the health care providers for treatment due to social problems, and (c) many husbands absolve themselves of their responsibility by only agreeing to pay for the treatment; thus affecting the treatment-seeking for GMs [46]. Contrary to past studies that found that health insurance leads to higher medical check-ups [47] and cervical screening among reproductive-age women [48], this study found no significant association between health insurance and treatment-seeking for GM among older adult women. This may be due to the perception that the problems are usual during older ages, so treatment is not required [10]. Another research found that the most prevalent cause for women not seeking treatment for GM was their belief that they did not require treatment [49]. In conformity to a past study, we also found higher treatment seeking among women from the southern region [50]. This may result from better health infrastructure and elevated female literacy levels in the southern region compared to the other region. As found in an earlier study [51], we also noticed lower treatment-seeking among women with no/less education and lower economic status. Our results reveal lower treatment-seeking among socio-economically disadvantaged groups—which calls for the urgent need to develop strategies to address these vulnerabilities and inequities.

There are several strengths of this study. To the best of our knowledge, it is the first study to analyze the prevalence and determinants of GM and treatment-seeking behavior of older adult women using a nationally representative data. Secondly, this study uses the recent large-scale LASI data with a robust sampling design; thus, the results are contemporary and relevant. Nevertheless, the results are based on cross-sectional data, so inferences drawn on the causal association between the predictor and outcome variables should be carefully studied. GMs are self-reported; thus, the possibility of under-reporting cannot be ruled out. Treatment-seeking for GM may also be influenced by several cultural and contextual factors, which this study could not include due to the unavailability of the survey data.

### Implications for policy and practice

The study reemphasizes the need for a life-course approach in women’s health in general and SRH in particular in the Indian context. The strategies under NHP aimed at enhanced provisions for reproductive morbidities and health needs of women beyond the reproductive age group should be rigorously implemented and monitored. Existing policies and programs should target the more vulnerable section for GMs, such as women of higher parity and those who have undergone hysterectomies. Lower treatment-seeking suggests a need for more awareness of the adverse implications of GMs, which may be addressed by engaging the grassroots community and health workers in delivering health messages to older adults. An earlier study also suggests the engagement of community-based health workers to improve health-seeking for multi-morbidity among older adults beyond reproductive age in India [52]. Efforts to sensitize women through community-based activities and awareness camps may reduce the stigma associated with GM among older adults and enhance their health-seeking for GM.

## Conclusion

Many older adult women had GM, and treatment-seeking was inadequate. The GM prevalence and treatment-seeking vary considerably by socioeconomic and demographic characteristics. Results suggest awareness generation and the inclusion of this otherwise ignored group in existing and future programs targeting better health and wellbeing of women. Improved health of older adult women will contribute to achieving Goals 3 and 5 of the Sustainable Development Goals (SDGs).

## Data Availability

This study was conducted by the MoHFW and the International Institute for Population Sciences (IIPS) in India. Ethical standards being followed, including informed consent being obtained by all participants. The data of the Longitudinal Ageing Study in India (LASI-Wave 1), 2016–2017 is available on request @ https://iipsindia.ac.in/content/data-request.
